# MicroRNA Profile of MA-104 Cell Line Associated With the Pathogenesis of Bovine Rotavirus Strain Circulated in Chinese Calves

**DOI:** 10.3389/fmicb.2022.854348

**Published:** 2022-04-11

**Authors:** Gehad Elkady, Yingyu Chen, Changmin Hu, Jianguo Chen, Xi Chen, Aizhen Guo

**Affiliations:** ^1^The State Key Laboratory of Agricultural Microbiology, Huazhong Agricultural University, Wuhan, China; ^2^College of Veterinary Medicine, Cooperative Innovation Centre of Substantial Pig Production, Huazhong Agricultural University, Wuhan, China; ^3^Benha University, Benha, Egypt; ^4^Hubei Hongshan Laboratory, Huazhong Agricultural University, Wuhan, China; ^5^Hubei International Scientific and Technological Cooperation Base of Veterinary Epidemiology, Huazhong Agricultural University, Wuhan, China

**Keywords:** bovine rotavirus G8P[7] isolate, miRNA, deep sequencing, miRNA-mRNA interaction, signaling pathway

## Abstract

Bovine rotavirus (BRV) causes massive economic losses in the livestock industry worldwide. Elucidating the pathogenesis of BRV would help in the development of more effective measures to control BRV infection. The MA-104 cell line is sensitive to BRV and is thereby a convenient tool for determining BRV–host interactions. Thus far, the role of the microRNAs (miRNAs) of MA-104 cells during BRV infection is still ambiguous. We performed Illumina RNA sequencing analysis of the miRNA libraries of BRV-infected and mock-infected MA-104 cells at different time points: at 0 h post-infection (hpi) (just after 90 min of adsorption) and at 6, 12, 24, 36, and 48 hpi. The total clean reads obtained from BRV-infected and uninfected cells were 74,701,041 and 74,184,124, respectively. Based on these, 579 were categorized as known miRNAs and 144 as novel miRNAs. One hundred and sixty differentially expressed (DE) miRNAs in BRV-infected cells in comparison with uninfected MA-104 cells were successfully investigated, 95 of which were upregulated and 65 were downregulated. The target messenger RNAs (mRNAs) of the DE miRNAs were examined by bioinformatics analysis. Functional annotation of the target genes with Gene Ontology (GO) and Kyoto Encyclopedia of Genes and Genomes (KEGG) suggested that these genes mainly contributed to biological pathways, endocytosis, apoptotic process, trans-Golgi membrane, and lysosome. Pathways such as the mammalian target of rapamycin (mTOR) (mml-miR-486-3p and mml-miR-197-3p), nuclear factor kappa B (NF-κB) (mml-miR-204-3p and novel_366), Rap1 (mml-miR-127-3p), cAMP (mml-miR-106b-3p), mitogen-activated protein kinase (MAPK) (mml-miR-342-5p), T-cell receptor signaling (mml-miR-369-5p), RIG-I-like receptor signaling (mml-miR-504-5p), AMP-activated protein kinase (AMPK) (mml-miR-365-1-5p), and phosphatidylinositol-3-kinase/protein kinase B (PI3K/Akt) signaling (mml-miR-299-3p) were enriched. Moreover, real-time quantitative PCR (qPCR) verified the expression profiles of 23 selected DE miRNAs, which were consistent with the results of deep sequencing, and the 28 corresponding target mRNAs were mainly of regulatory pathways of the cellular machinery and immune importance, according to the bioinformatics analysis. Our study is the first to report a novel approach that uncovers the impact of BRV infection on the miRNA expressions of MA-104 cells, and it offers clues for identifying potential candidates for antiviral or vaccine strategies.

## Introduction

Rotaviruses (RVs) account for the severe diarrhea in children and young animals globally. RVs belong to the genus *Rotavirus*, which is one of the 15 genera of the Reoviridae family (Matthijnssens et al., 2012). They are non-enveloped, double-stranded, and segmented RNA viruses. The 11 segments encode 11 or 12 viral proteins, including six structural proteins (VPs) and five or six non-structural proteins (NSPs) (Estes and Greenberg, 2013). Based on *VP6*, genetically and antigenically, RVs are categorized into 10 groups (species A–J) (Bányai et al., 2017). Besides being a disease cause, species A rotaviruses (RVAs) have a large economic impact on human and animal species (Matthijnssens et al., 2010). Based on the antigenic features of VP4 and VP7, RVs are further classified into the G and P genotypes and serotypes, respectively (Estes and Greenberg, 2013). MicroRNAs (miRNAs) are a unique class of small RNAs (sRNAs), which are endogenous, single-stranded, non-coding RNAs (ncRNAs) of 19–24 nucleotides (nt) in length, expressed by all multicellular eukaryotes. This class has demonstrated significant evolutionary conservation (Friedman et al., 2009), which can inhibit the expressions of complementary target messenger RNAs (mRNAs) *via* transcriptional destabilization or translational inhibition (Bartel, 2009). Many studies have demonstrated that miRNAs are involved in a variety of biological processes, such as cell differentiation, development, homeostasis, proliferation, apoptosis, and signal transduction, in animals and plants (Axtell et al., 2011). RNA virus infection can mediate changes in the expression of the host miRNA machinery, leading to downstream changes in the host transcriptome that can be favorable to viral replication and pathogenesis. Notwithstanding, these changes in miRNA expression can also lead to increases in antiviral effector activities, resulting in decreased viral replication (Trobaugh and Klimstra, 2017). Mature miRNA guides RNA-induced silencing complexes (RISCs) to mRNAs bearing complementary target sites (Hammond et al., 2000). RISCs, which are minimally composed of a mature miRNA and an Argonaute protein, usually, but not invariably, bind to the 3′-untranslated region (UTR) of the targeted transcripts. Functional targets are generally fully complementary to nucleotides 2–7, preferably 2–8, at the 5′ end of the miRNA, referred to as the miRNA seed (Bartel, 2009). However, there are a few examples of base pairing between the target transcript and the 3′ half of the miRNA that can compensate for mismatches in the seed. Perhaps the best example of this phenomenon was provided by the miRNA lin-4 and the well-characterized target mRNA encoding lin-14, where robust inhibition of the expression of lin-14 was conferred by target sites lacking full seed homology (Ha et al., 1996). A single miRNA can target multiple mRNAs, and a single mRNA 3′-UTR can contain binding sites for several miRNAs (Bartel, 2009). The genes for miRNAs are distributed in different genomic locations, such as the intergenic regions, introns, and exons of protein-coding genes. Most miRNA genes are transcribed individually and scattered across the entire genome, whereas a portion of these genes is clustered and transcribed as one long primary miRNA (pri-miRNA) transcript and processed into individual precursor miRNAs (pre-miRNAs) (Altuvia et al., 2005). According to miRBase, humans encode >500 different miRNAs (Griffiths-Jones et al., 2008), which tend to be expressed in a developmental or tissue-specific manner (Landgraf et al., 2007). It has been demonstrated that individual miRNAs are capable of directly downregulating the expressions of hundreds of different mRNAs, and >30% of all mammalian mRNAs are thought to be regulated by miRNAs (Lewis et al., 2005). Some studies have characterized cellular miRNAs after infection with human immunodeficiency virus (HIV) (Chang et al., 2013), hepatitis C virus (HCV) (Norman and Sarnow, 2010), influenza virus (IV) (Skovgaard et al., 2013), West Nile virus (WNV) (Slonchak et al., 2014), Japanese encephalitis virus (JEV) (Zhang et al., 2015), dengue fever virus (DENV) (Liu et al., 2015), bluetongue virus (BTV) (Xing et al., 2016), and vesicular stomatitis virus (VSV) (Otsuka et al., 2007). Notably, one study reported on a comprehensive miRNA analysis of HT29 and MA104 cells infected with the human RV strain ZTR-68 (P[8] G1) (Zhou et al., 2016) and of Caco-2 cells infected with the human RV strain Wa (Tian et al., 2017). Here, our research aimed at analyzing the differentially expressed (DE) miRNAs of host cells before and after bovine rotavirus (BRV) infection. Data on the differential miRNAs induced by the interaction between BRV and host cells could fill the gap in viral pathogenesis, host innate antiviral defense, and viral replication and thus can be used for future development of novel antiviral drugs and vaccines against this virus.

## Materials and Methods

### Virus and Cell Culture

The BRV strain G8P[7] (RVA/Cow-tc/CHN/10927/2019/G8P[7]) was previously isolated from Chinese diarrheal calves in MA-104 cells and characterized by mass spectrometry. The complete protein sequences of the 11 segments were deposited to the ProteomeXchange Consortium *via* the PRIDE partner repository with the dataset identifier PXD017047 (Elkady et al., 2021). The accession numbers in the Uniprot database are provided in Supplementary Table 1. The viral titer calculated using the Reed and Muench equation (Reed and Muench, 1938) was 10^4.5^ TCID_50_/50 μl. The MA-104 cell line was purchased from Procell Life Sciences and Technology Co., Ltd. (Wuhan, China). The cells were sub-cultured in six-well tissue culture plates (cat. no. 002019; Corning, Inc., Corning, NY, United States). Dulbecco’s modified Eagle’s media (DMEM) (cat. no. 11965092; Gibco™, Waltham, MA, United States) complemented with 5%–10% fetal bovine serum (cat. no. 26140-079; Gibco™) and antibiotics (1,000 IU penicillin and 1,000 μg/ml streptomycin) (cat. no. 15140122; Sigma-Aldrich, St. Louis, MO, United States) were used.

### Virus Infection

Cells were used for BRV infection when grown to 80–90% confluence. They were infected with 100 TCID_50_ of the BRV strain G8P[7]. Uninfected cells were used as the mock-infected group. The cytopathic effect (CPE) of the infected cells was observed at 0, 6, 12, 24, 36, 48, and 72 h post-infection (hpi) in comparison with uninfected cells. Virus propagation and the associated CPE were confirmed by RT-PCR of the *VP6* gene (primers: F, 5′-ACCACCAAATATGACACCAGC-3′; R, 5′-CATGCTTCTAATGGAAGCCAC-3′) (Elkady et al., 2021). The PCR products were sequenced and confirmed using an automated sequencer (ABI3730, Applied Biosystems, Bedford, MA, United States; TSINGKE Biological Technology, Beijing, China).

### Detection of Rotavirus dsRNAs by SDS–PAGE

To further detect and confirm the propagation of BRV in the culture fluid, the culture flask that showed typical CPE at 72 hpi was completely frozen at −80°C and then completely thawed in one cycle at 4°C. Thereafter, total RNA was extracted from an almost 4-ml infected cellular fluid using the TRIzol reagent (15596-026; Ambion^®^, Life Technologies, Carlsbad, CA, United States) according to the manufacturer’s instruction. The extracted RNA was loaded into an SDS–PAGE 2 × loading buffer. The loaded RNA was screened by 12% resolving polyacrylamide gel using a vertical electrophoresis apparatus and at 80 V, 3 h, 20 mA (two glass plates of 1 mm thickness) (Bio-Rad, Hercules, CA, United States). Finally, the 11 segments of viral double-stranded RNA (dsRNA) were visualized with a silver stain and photographed (Gray and Desselberger, 2000).

### RNA Purification and Quality Control

The infected and control cell samples (three biological replicates) were collected from six-well plates for each of the following time points: 0 hpi (just after 90 min adsorption) and at 6, 12, 24, 36, and 48 hpi. Three biological replicates of the virus- and mock-infected cultures were prepared at each time point. Total RNA was purified from the cells using the TRIzol reagent (15596-026; Ambion^®^, Life Technology). Then, strict quality control was carried out for the RNA samples. The quality control criteria included the following aspects: (1) agarose gel electrophoresis analyzed the integrity of sample RNA and excluded DNA contamination; (2) NanoDrop was used for preliminary quantification, detection of RNA concentration, and purity (OD_260/280_ and OD_260/230_); and (3) Agilent 2100 Bioanalyzer was used for accurate detection of RNA integrity.

### Small RNA Sequencing

**FIGURE 1:**
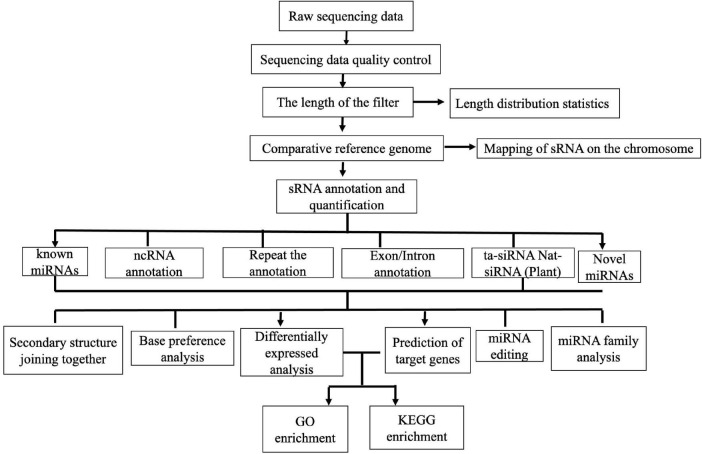
Parametric analysis process of animal small RNA.

The small RNA-seq process mainly consists of two parts: database-building sequencing and bioinformatics analysis. After detection of qualified samples, a Small RNA Sample Pre-Kit was used to construct the sRNA library. The special structure of the 3′ and 5′ ends of sRNA was used (the 5′ end had the complete phosphate group and the 3′ end had the hydroxyl group). Total RNA was used as the starting sample, and both ends of sRNA were directly added to the joint. It was then reversely transcribed into complementary DNA (cDNA). After PCR amplification, the target DNA fragments were separated by SDS–PAGE, and the cDNA library was recovered by gel cutting. After library construction, Qubit 2.0 was used for preliminary quantification, and the library was diluted to 1 ng/μl. Then, the highly sensitive Agilent 2100 was used to detect the insert size of the library. After meeting the insert size expectations, the effective concentration of the library was accurately quantified by quantitative PCR (qPCR) (effective library concentration, > 2 nM) to ensure quality. After qualified library inspection, different libraries were pooled into Illumina SE50 sequencing according to the requirements of effective concentration and target offline data amount. The reaction system was simultaneously added with DNA polymerase, coupling primers, and four dNTPs with base-specific fluorescence markers (as Sanger sequencing method). These dNTP 3′-OH groups were chemically protected so that only one dNTP can be added. After the addition of dNTP to the synthetic chain, all unused free dNTP and DNA polymerase were eluted. Then, the buffer was added for fluorescence excitation, the fluorescence signal was stimulated by laser, and it was recorded by an optical equipment. Finally, the optical signal was converted into the sequencing base by computer analysis. After fluorescence signal recording, chemical reagents were added to quench the fluorescence signal and remove the dNTP 3′-OH protective group, so that the next round of sequencing reaction was carried out. The Illumina technology, adding only one dNTP at a time, solves the issue of accurately measuring the length of the homomer. All sRNA families in the samples were sequenced and expressed quantitatively with a high-throughput sequencing technology, and the miRNA, small interfering RNA (siRNA), Piwi-interacting RNA (piRNA), and other non-coding RNAs, as well as the corresponding target sequences, were analyzed. The parametric analysis process of animal sRNA was adopted [quality control of the original sequencing data, statistics of clean read length distribution, sRNA annotation, known miRNA annotation, known piRNA annotation, ncRNA analysis, prediction of new miRNA (with reference genome), differential gene analysis, miRNA family analysis, cluster analysis of the DE miRNAs, prediction of miRNA target genes, and Gene Ontology (GO) and Kyoto Encyclopedia of Genes and Genomes (KEGG) enrichment analyses of the target genes] (Figure 1). The raw sequence data obtained by high-throughput sequencing were analyzed by base calling and transformed into sequenced reads, called raw data or raw reads. The results were stored in FASTQ format, which contains the sequence information of the sequencing sequences (reads) and their corresponding sequencing quality information. The clean reads of each sample were screened, sRNAs within a certain length were selected for subsequent analysis, and the length distribution of sRNAs was determined; the length range of animal sRNA is 18–35 nt (Bartel, 2018). The sRNAs were aligned and analyzed in comparison with the reference genome sequence in the Sanger miRBase 21.0 database^1^ (Langmead et al., 2009). The expression levels of the known and novel miRNAs in each sample were calculated, and TPM (transcript per million) was used to normalize the expression levels (Zhou et al., 2010). For the samples with biological duplication, DESeq2 (Love et al., 2014) based on negative binomial distribution was used for analysis. For samples without biological duplication, DEGseq (Wang et al., 2010) based on the TMM (trimmed mean of *M*-values) algorithm was used to standardize the read count data and then conduct difference analysis. The thresholds for differences of miRNA filter condition are: *p*_*adj*_ < 0.05 and | log2(fold change) | > 1. Additionally, the DE miRNAs between the different BRV infection time points were identified. Moreover, animal miRNA target genes were predicted using miRanda^2^ (Enright et al., 2003) and RNAhybrid^3^ (Krüger and Rehmsmeier, 2006).

### Enrichment Analysis of the Differential MicroRNAs Target Genes

After obtaining the DE miRNAs from each infection time point, we conducted GO and KEGG enrichment analyses for the collected DE miRNA target genes in each group according to the corresponding relationship between miRNA and its target genes. GO^4^, which is the international standard classification system for gene function, was used to study the function of the target mRNA. The GO enrichment analysis method used was GOseq (Young et al., 2010), based on Wallenius non-central hypergeometric distribution. In addition, KEGG (Kanehisa et al., 2008) was adopted to analyze the biochemical metabolic pathways and signal transduction pathways (protein--protein interaction) involving the target gene candidates; pathway significance enrichment analysis was conducted using KEGG PATHWAY (KOBAS 2.0 annotation tool)^5^. The hypergeometric test/Fisher’s exact test was used to determine which pathway was significantly enriched in the candidate target genes compared with the whole genome background. The calculation formula for this analysis is as follows:


$$p=1-\sum_{i=0}^{m-I}\frac{\left[\begin{array}{c} M\\ i \end{array}\right]\,\left[\begin{array}{c} N-M\\ n-i \end{array}\right]}{\left[\begin{array}{c} N\\ n \end{array}\right]}$$

where *N* is the number of genes with pathway annotation in all genes, *n* is the number of candidate target genes in *N*, *M* is the number of genes annotated as a specific pathway in all genes, and *m* is the number of candidate target genes annotated as a specific pathway. For the false discovery rate (FDR) correction method, Benjamini–Hochberg (BH) was used for *p*-Value correction; the smaller the corrected *p*-Value, the more significant it is. Pathways with values less than 0.05 were defined as significantly enriched in candidate target genes. KEGG enrichment was measured using Rich factor, *Q*-Value, and the number of genes enriched in a pathway. Rich factor refers to the ratio of the number of genes in the DE genes to the total number of genes in the pathway item in all the annotated genes. The larger the Rich factor, the greater the degree of enrichment. The *Q*-value is the *p*-Value after multiple hypothesis testing and correction. The range of *Q*-values is [0, 1]; the closer the value is to zero, the more significant the enrichment.

### Real-Time Relative Quantitative PCR

We selected some DE miRNAs (*n* = 23) and their target genes (*n* = 28) for validation by real-time relative qPCR based on the following criteria: (1) they were previously reported to modulate the cellular machinery (apoptosis, autophagy, cancer, and immunity) with their corresponding target mRNAs of immune importance; (2) they were in the top 20 significantly enriched cellular signaling pathways; (3) some novel DE miRNAs with the highest differential folds; and (4) some of the corresponding target mRNAs associated with pathways responsible for host antimicrobial response. Total RNAs at different time points—0 hpi (just after 90 min adsorption) and 6, 12, 24, 36, and 48 hpi—from the virus-infected and non-infected groups were extracted and purified. They were then reverse transcribed to cDNA using miRNA 1st-Strand cDNA Synthesis Kit (by stem–loop) (#MR101; Vazyme, Nanjing, China), according to the manufacturer’s instruction, using the RT-specific primer. The gene expression and the differential expression of the miRNAs were determined with the miRNA Universal SYBR^®^ qPCR Master Mix (#MQ101; Vazyme), following the manufacturer’s instruction, using specific reverse and forward primers. *U6* was used as an internal reference gene. In addition, for the detection of the DE target mRNAs, RNA was reverse transcribed into single-strand cDNA using the Transcriptor First Strand cDNA Synthesis Kit (cat. no. 04897030001; Roche, Basel, Switzerland) according to the manufacturer’s instructions. The gene expression and the differential expression of the target mRNAs were determined using the AceQ^®^ qPCR SYBR Green Master Mix (#Q111; Vazyme). *β-actin* was used as an internal reference gene. The primers used in the reverse transcription and real-time qPCR reactions are listed in Supplementary Table 2. All qPCR reactions were performed using Bio-Rad CFX-96. The relative normalized expressions of the DE miRNAs and their target mRNAs were calculated with the 2^–ΔΔ*CT*^ method using Bio-Rad software data analysis from three independent experiments. The gene regulation threshold > 2 and an FDR-adjusted *p*-Value < 0.05 were considered as the parameters for statistical significance of the DE genes.

### Statistical Analysis

Unpaired, two-tailed Student’s *t*-test for grouped data was used with GraphPad Prism 8 software, and comparative analysis of the fold change (FC) values (2^–ΔΔ*CT*^) between the non-infected and virus-infected groups was performed. All qPCR data are presented as the mean ± SD and *p* < 0.05 was considered statistically significant. In addition, sequencing data above the thresholds were screened using default statistical analysis, which was explained in the methodological description.

## Results

### Confirmation of Bovine Rotavirus Replication in MA-104 Cells

**FIGURE 2:**
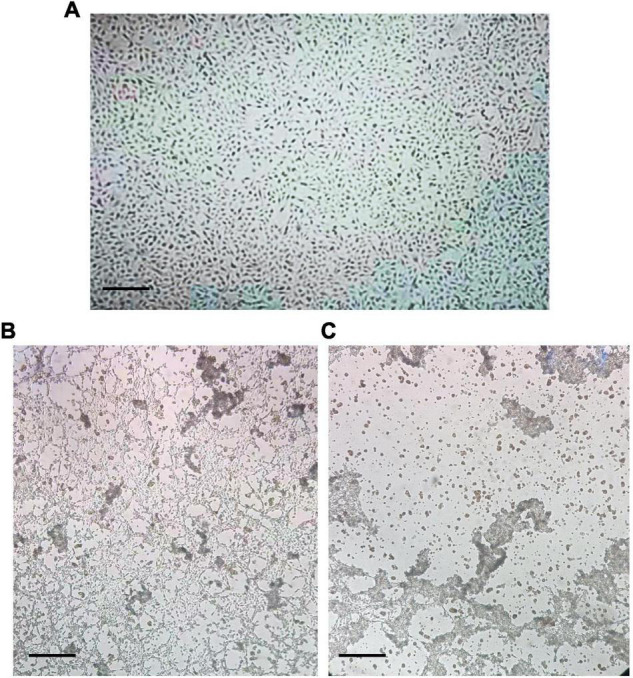
Morphological observation of the cytopathic effect (CPE) of bovine rotavirus (BRV) in MA-104 cells. **(A)** Uninfected MA-104 cells with an intact cell sheet. **(B)** CPE of infected MA-104 cells at 48 h post-infection (hpi) with BRV. Partial cells in the cell sheet became lysed and were detached from the growth surface. **(C)** CPE of infected MA-104 cells at 72 hpi. The cells showed complete lysis and detachment from the growth surface. *Bar* indicates × 4 magnification.

**FIGURE 3:**
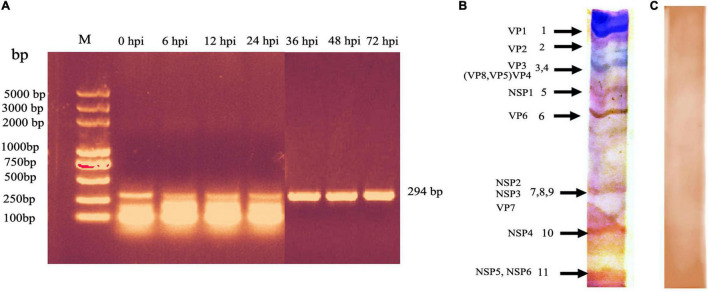
PCR detection of the bovine rotavirus (BRV) isolate *VP6* gene during the course of the virus inside the cell after infection (0–72 hpi) and detection of the 11 genome segments of BRV by SDS–PAGE. **(A)** Amplicon of the *VP6* gene segment (294 bp). *M*: DNA ladder DL5000bp; *lanes 1*–*7*, samples from cell culture harvest. The bands were visualized in 1% TS-GelRed stain in agarose gel. **(B)** RNA profile of group A BRV isolate. Typical RNA migration pattern showed that segments *7*, *8*, and *9* very close to each other, producing one segment, while segments *3* and *4* appeared as one segment. **(C)** Negative control of the SDS–PAGE image.

Compared with the mock-infected (control) cells, no CPE was observed in the BRV-infected MA-104 cells from 0 to 36 hpi (Figure 2A), whereas the typical CPE of BRV appeared within 48 and 72 hpi (Figures 2B,C). To confirm viral replication in MA-104 cells following BRV infection, the cell-associated viruses were harvested before and after the occurrence of CPE and were confirmed *via* RT-PCR to amplify the viral (*VP6*) gene (accession. no MW018712) (Figure 3A). Sequencing analysis of the PCR products further validated BRV infection. Additionally, it was confirmed by the typical RNA pattern of this group A BRV strain, G8P[7], in comparison with the negative control using SDS–PAGE and silver stain (Figures 3B,C).

### MicroRNA Expression and Differential Expression

**FIGURE 4:**
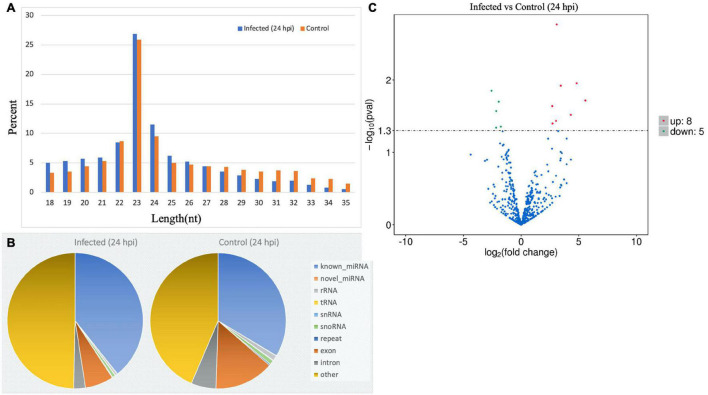
MicroRNA (miRNA) expression profiles of bovine rotavirus (BRV)-infected cells in comparison with the control (24 hpi). **(A)** Length distributions of total small RNA (sRNA) segments: the length of reads proportional to the reads of that length (23 nt was the main observed length). **(B)** Pie chart showing the total read distributions of sRNAs and their categories (e.g., known miRNAs, novel miRNAs, rRNA, tRNA, snRNA, snoRNA, repeats, exons, introns, etc.). **(C)** Volcano chart representing the expression change of the miRNAs between the virus-treated and normal non-treated cells. The *x*-axis shows the log2(fold change) and the *y*-axis shows the −log10 (*q*-Value). The *horizontal dotted lines* in the figure correspond to the *q*-Value (default) or the adjusted *p*-Value (FDR), with 0.05 as the significant difference threshold. Significant difference thresholds: log2(fold change) > 1 and FDR-adjusted *p* = 0.05. Upregulated miRNAs are represented by *red dots*, downregulated miRNAs by *green dots*, and miRNAs with no significant changes by *blue dots*.

RNAs of grade A quality were used for sequencing and differential analysis of the virus-treated and non-infected MA-104 cells in triplicate. Raw reads from the libraries derived from the virus-treated and control cell groups at different time points after infection (0–48 hpi) were generated. Then, the clean reads were obtained after filtration and sRNAs within a certain length were selected for subsequent analysis, as shown in Supplementary Tables 3a,b. The obtained length of the clean reads was 22–24 nt, mainly with the length of 23 nt at 24 hpi (Figure 4A) and at 0, 6, 12, 36, and 48 hpi (Supplementary Figures 1a–e). The percentages of non-coding nucleic acids [e.g., known miRNAs, novel miRNAs, ribosomal RNA (rRNA), transfer RNA (tRNA), snRNA, small nucleolar RNA (snoRNA), repeats, exons, introns, etc.] in the virus-infected and control groups were shown at 24 hpi (Figure 4B) and at 0, 6, 12, 36, and 48 hpi (Supplementary Figures 2a–e). The mapped known and novel miRNAs of *Macaca mulatta* species are listed in Supplementary Table 4. After BLASTN analysis by miRBase 20.0 with *M. mulatta* miRNAs, 579 known and 144 novel mature miRNAs were obtained. The DE miRNAs with true significance (FDR < 0.05 and log2 FC > 1) at the different time points were identified. Basically, the DE miRNAs, in both number and genes, varied with time post-infection, including 20 upregulated and 10 downregulated at 0 hpi, 11 upregulated and 2 downregulated at 6 hpi, 8 upregulated and 14 downregulated at 12 hpi, 8 upregulated and 5 downregulated at 24 hpi, 27 upregulated and 23 downregulated at 36 hpi, and 21 upregulated and 11 downregulated at 48 hpi (Supplementary Tables 5a–f). However, some common DE miRNAs exist between the different time points of BRV infection, such as mml-miR-99a-5p between 0 and 6 hpi, mml-miR-342-5p between 12 and 24 hpi, novel_458 between 12 and 36 hpi, mml-miR-411-3p between 12 and 48 hpi, novel_356 and novel_66 between 24 and 36 hpi, and mml-miR-411-5p between 36 and 48 hpi (shown in Figure 4C for 24 hpi and in Supplementary Figures 3a–e for 0, 6, 12, 36, and 48 hpi).

### Interactions Between MicroRNAs and Messenger RNAs

**FIGURE 5:**
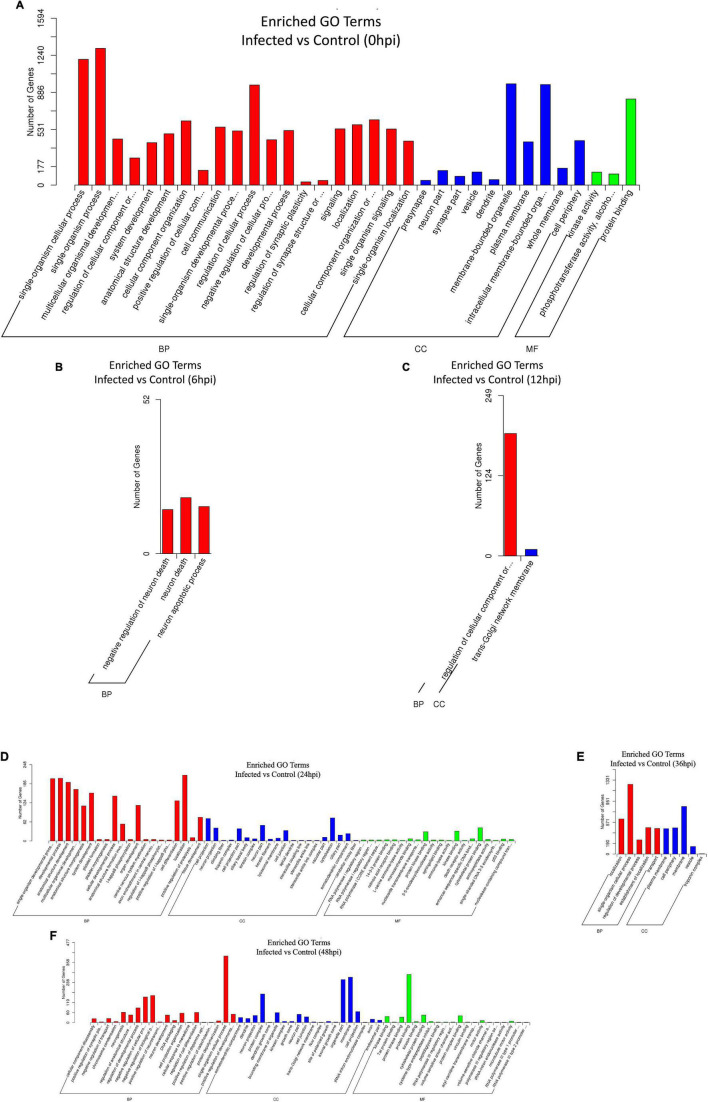
**(A–F)** Gene Ontology (GO) enrichment histogram of candidate target genes between the infected and control groups from 0 to 48 hpi. The proportions of the three categories of GO [biological process (BP), cellular component (CC), and molecular function (MF)] and the number of candidate target genes annotated are shown.

**FIGURE 6:**
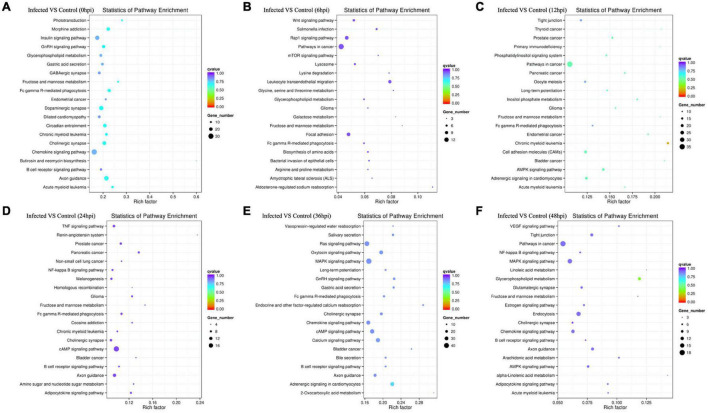
**(A–F)** Rich distribution points of candidate target genes with Kyoto Encyclopedia of Genes and Genomes (KEGG) enrichment between the infected and control groups from 0 to 48 hpi. The *vertical axis* represents the pathway name, the *horizontal axis* represents the Rich factor, the *size of the dots* indicates the number of candidate target genes in this pathway, and the *color of the dots* corresponds to the different *Q*-value ranges.

All target genes and their specific mRNA transcripts in the known and novel miRNAs were bioinformatically expected, and the corresponding relationship between the DE miRNA and the target genes was characterized (Supplementary Table 6a). All annotated target mRNAs are listed in Supplementary Table 6b. GO and KEGG analyses were conducted to characterize the functions of the predicted target mRNAs of the DE miRNAs. The difference in probability was estimated based on the bias of gene length so that the probability of the GO term being enriched by the candidate target genes can be more accurately calculated. The GO enrichment list of the candidate target genes associated with the different infection times is shown in Supplementary Tables 7a–f. Mostly, the most significantly enriched GO terms of the target mRNAs between the infected and control cell groups were involved in biological process (BP), cellular component (CC), and molecular function (MF) at 0, 6, 12, 24, 36, and 48 hpi (Figures 5A–F). At 0 hpi, the targets included in functions such as membrane-bound organelle and plasma membrane could help the virus to attach to the cellular receptor and internalize the cells; the target mRNAs were mainly associated with the apoptotic process at 6 hpi and contributed to the trans-Golgi membrane at 12 hpi. Evidently, the target genes produced at 24 hpi played a role in polymerase activity and genome replication, such as RNA polymerase I regulatory region, RNA polymerase I core element sequence, and binding proteins. Moreover, the targets were involved in the localization and regulation of developmental process at 36 hpi and in DNA packaging, trans-Golgi network membrane, and polymerase III regulatory regions at 48 hpi. On the other hand, all the enriched pathways characterized through KEGG mapping were shown (Supplementary Tables 8a–f), highlighting the top 20 significantly enriched pathways represented by the predicted target mRNAs of each comparison group (Figures 6A–F and Supplementary Tables 9a–f). KEGG enrichment elucidated the pathways that played a part in the pathogenesis of the virus, such as the B-cell receptor signaling pathway, Fc gamma R-mediated phagocytosis, and chemokine signaling pathway at 0 hpi; pathways in cancer, Rap1 signaling pathway, mTOR signaling pathway, Wnt signaling pathway, lysosome, leukocyte trans-endothelial migration, lysine degradation, and Fc gamma R-mediated phagocytosis at 6 hpi; and pathways in cancer, MAPK signaling, primary immunodeficiency, acute myeloid leukemia, cell adhesion molecules (CAMs), long-term potentiation, and Fc gamma R-mediated phagocytosis at 12 hpi. In addition, at 24 hpi, the cAMP, NF-κB, and TNF signaling pathways, Fc gamma R-mediated phagocytosis, B-cell receptor signaling pathway, and chronic myeloid leukemia were involved. On the other hand, the MAPK and Ras signaling pathways, Fc gamma R-mediated phagocytosis, long-term potentiation, and B-cell receptor signaling pathway were included at 36 hpi. Last but not least, pathways in cancer, MAPK signaling, the NF-κB and B-cell receptor signaling pathway, endocytosis, AMPK signaling pathway tight junction, arachidonic acid metabolism, and the chemokine signaling pathway were implicated at 48 hpi.

### Validation of Differentially Expressed MicroRNAs and Target Messenger RNAs

**FIGURE 7:**
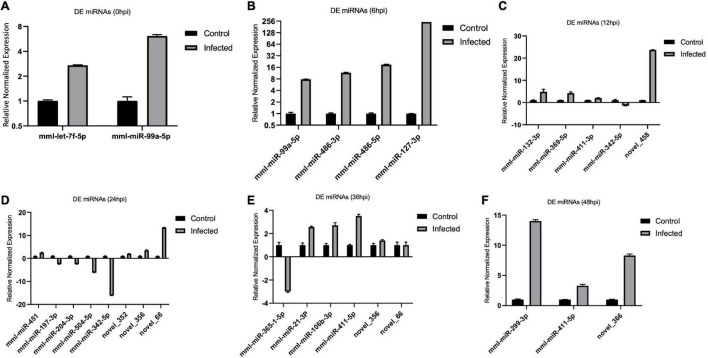
**(A–F)** Validation of the differentially expressed (DE), microRNAs (miRNAs) by real-time quantitative PCR (qPCR) from 0 to 48 hpi. The differences in expression were determined using the 2^– ΔΔCT^ method and with unpaired, two-tailed Student’s *t*-test for grouped data. All experiments were performed in triplicate (*n* = 3). *U6* was used as an internal reference. Data represent the mean ± SD (*error bar*). Significant difference thresholds: log2(fold change) > 2 and FDR-adjusted *p* < 0.05.

**FIGURE 8:**
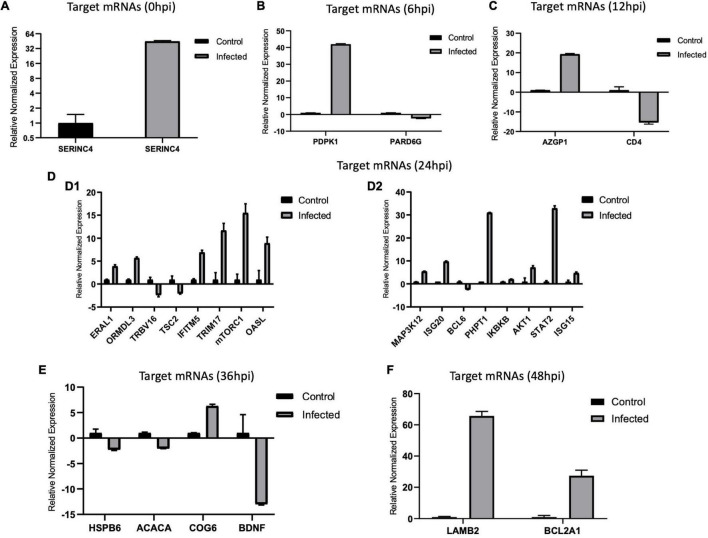
**(A–F)** Validation of target messenger RNAs (mRNAs) by real-time quantitative PCR (qPCR) from 0 to 48 hpi. The differences in expression were determined using the 2^– ΔΔ*CT*^ method and with unpaired, two-tailed Student’s *t*-test for grouped data. All experiments were performed in triplicate (*n* = 3). *β-actin* was used as an internal reference. Data represent the mean ± SD (*error bar*). Significant difference thresholds: log2(fold change) > 2 and of FDR-adjusted *p* < 0.05.

**FIGURE 9:**
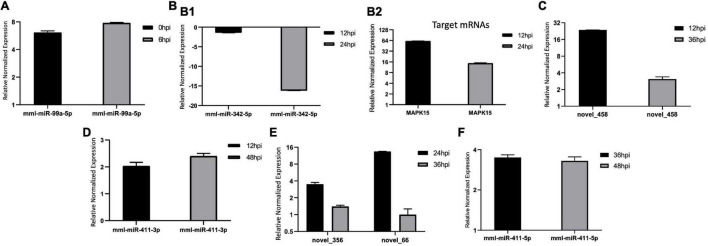
**(A–F)** Expression levels of the common differentially expressed (DE) microRNAs (miRNAs) at different time points of bovine rotavirus (BRV) infection. The differences in expression were determined using the 2^– ΔΔ*CT*^ method and with unpaired, two-tailed Student’s *t*-test for grouped data. All experiments were performed in triplicate (*n* = 3). *U6* was used as an internal reference. Data represent the mean ± SD (*error bar*). Significant difference thresholds: log2(fold change) > 2 and FDR-adjusted *p* < 0.05.

We validated the DE miRNAs (*n* = 23) and their corresponding target mRNAs (*n* = 28) at 0–48 hpi. The expression change values of all validated miRNAs and genes were in agreement with those in the deep sequencing and the bioinformatics analyses. Some of the DE miRNAs showed significant upregulation, such as mml-let-7f-5p (0 hpi; FC = ∼2.7, *p* = ∼0.00004) and its target (*SERINC4*; FC = ∼45, *p* = ∼0.001871), mml-miR-486-3p (6 hpi; FC = ∼11.5, *p* = ∼0.000011) and its target (*PDPK1;* FC = ∼42, *p* = ∼0.02), and mml-miR-486-5p (at 6 hpi; FC = ∼18.8, *p* = ∼0.001). Interestingly, mml-miR-127-3p (6 hpi; FC = ∼242.5, *p* = ∼0.000002) and its target (*PARD6G*; FC ∼ −2.3, *p* = ∼0.02) showed very high upregulation. In addition, mml-miR-132-3p (12 hpi; FC = ∼4.8, *p* = ∼0.03666) and its target (*AZGP1*) were dramatically upregulated (FC = ∼19.34, *p* = ∼0.0068). Moreover, mml-miR-369-5p (12 hpi) was upregulated (FC = ∼4.2, *p* = ∼0.003), and its target (*CD4*) was sharply downregulated with (FC ∼ −15.3, *p* = ∼0.000719). Additionally, mml-miR-451 (24 hpi; FC = ∼2.06, *p* = ∼0.040658) and its target *ERAL1* (FC = ∼3.9, *p* = ∼0.01) were upregulated, and novel_352 (24 hpi) showed FC = ∼2 (*p* = ∼0.05). Meanwhile, mml-miR-21-3p (36 hpi) exhibited FC = ∼2.56 (*p* = ∼0.000476), and its target (*COG6*) was upregulated (FC = ∼6.29, *p* = ∼0.001113). Also, mml-miR-106b-3p (36 hpi; FC = ∼2.7, *p* = ∼0.0008) and its target (*BDNF*; FC ∼ −13, *p* = ∼0.007) and mml-miR-299-3p (48 hpi; FC = ∼14, *p* = ∼0.0003) and its target (*LAMB2*; Fc = ∼65.7, *p* = ∼0.000097) were dramatically upregulated, while novel_366 (48 hpi) and its target (*BCL2A1*) showed values of FC = ∼8.3 (*p* = ∼0.02) and FC = ∼27.5 (*p* = ∼0.003), respectively. mml-miR-197-3p (24 hpi; FC ∼ −2.01, *p* = ∼0.005337) and its two targets, *TSC2* (FC ∼ −2.05, *p* = ∼0.04) and *TRBV16* (FC ∼ −2.39, *p* = ∼0.05), exhibited downregulation; however, its other target (*ORMDL3*) was dramatically upregulated (FC = ∼5.7, *p* = ∼0.003546). Moreover, mml-miR-204-3p (24 hpi) showed FC ∼ −2.47 (*p* = ∼0.012433), and most of its targets were upregulated: *IFITM5* (FC = ∼ = 6.9, *p* = ∼0.027985, *TRIM17* (FC = ∼11.7, *p* = ∼0.000946), *mTORC1* (FC = ∼15.5, *p* = ∼0.002), *OASL* (FC = ∼8.9, *p* = ∼0.000035), *MAP3K12* (FC = ∼5.5, *p* = ∼0.000283), *ISG20* (FC = ∼9.8, *p* = ∼0.003473), *PHPT1* (FC = ∼31, *p* = ∼0.000043), *IKBKB* (FC = ∼2.12, *p* = ∼0.000007), and *AKT1* (FC = ∼7.2, *p* = ∼0.000028). However, its target *BCL6* was downregulated (FC ∼ −2.5, *p* = ∼0.02). mml-miR-504-5p (24 hpi) was also downregulated (FC ∼ −6.2, *p* = ∼0.000007), and its two targets were upregulated: *STAT2* (FC = ∼32.9, *p* = ∼0.000005) and *ISG15* (FC = ∼4.8, *p* = ∼0.006). Additionally, at 36 hpi, mml-miR-365-1-5p showed a downregulation (FC ∼ −2.9, *p* = ∼0.004), along with its targets: *HSPB6*; FC ∼ −2.3, *p* = ∼0.0002) and *ACACA* (FC ∼ −2.08, *p* = ∼0.000263). Tables 1a,b list the DE miRNAs whose targets were implicated in the signaling pathways, while the DE miRNAs without targets or without significant targets and not implicated in the signaling pathways, confirmed by qPCR [marked with (−)] are shown in Figures 7A–F, 8A–F and in Supplementary Table 10. Additionally, the expression levels of the common DE miRNAs between the different time points of infection were analyzed and confirmed. The miRNA mml-miR-99a-5p was upregulated at 0 and 6 hpi (FC = ∼6.11, *p* = ∼0.011099 and FC = ∼7.79, *p* = ∼0.001, respectively). At 36 hpi, mml-miR-411-5p had FC = ∼3.5, *p* = ∼0.003, and it showed an almost the same expression level at 48 hpi (FC = ∼3.3, *p* = ∼0.000735). Similarly, the expression pattern of mml-miR-411-3p at 12 hpi (FC = ∼2.03, *p* = ∼0.04) was close to that at 48 hpi (FC = ∼2.4, *p* = ∼0.002). mml-miR-342-5p had FC ∼ −1.4 (*p* = ∼0.2) at 12 hpi; however, at 24 hpi, it showed a surge in fold change, with FC ∼ −16.2 (*p* = ∼0.000002), with its target *MAPK15* (12 hpi: FC = ∼61.5, *p* = ∼0.009; 24 hpi: FC = ∼14.7, *p* = ∼0.000222). Besides, novel_66 experienced very high regulation at 24 hpi (FC = ∼13.4, *p* = ∼0.0009), which dramatically dropped at 36 hpi (no fold change and *p*-Value). Moreover, novel_458 (12 hpi: FC = ∼23.7, *p* = ∼0.000111) showed a decrease to FC = ∼3.1 (*p* = ∼0.001) at 36 hpi, as well as novel_356 (24 hpi: FC = ∼3.5) decreasing to FC = ∼1.4 at 36 hpi, both without *p*-Value (Figure 9, Table 2, and Supplementary Table 11).

**TABLE 1a:** The selected DE miRNAs and their corresponding genes, corresponding target mRNA transcripts and the signaling pathways they implicated in.

DE miRNA	Illumina RNA Sequencing		qPCR		Corresponding target gene ID	Corresponding target gene name	Corresponding target mRNA ID	qPCR		KEGG signaling pathway
	(log2fold change)	*P*-value	(log2fold change)	*P*-value				(log2 fold change)	*P*-value	
mml-miR-486-3p (6 hpi)	6.25	3.803553301 36931E-13	11.5	0.000011	ENSMMUG000000 11459	PDPK1	ENSMMUT000000 58708	42	0.02	mTOR signaling pathway
mml-miR-127-3p (6 hpi)	2.4	0.00357868185 018267	242.5	0.000002	ENSMMUG000000 39029	PARD6G	ENSMMUT000000 67376	-2.3	0.02	Rap1 signaling pathway
mml-miR-369-5p (12 hpi)	1.2	0.005	4.2	0.003	ENSMMUG000000 13202	CD4	ENSMMUT000000 18518	-15.3	0.000719	Primary immune deficiency, T cell receptor signaling pathway
mml-miR-197-3p (24 hpi)	-1.7	0.04	-2.5	0.005337	ENSMMUG0000000 2808	TSC2	ENSMMUT000000 59008	-2.05	0.04	mTOR signaling pathway
mml-miR-204-3p (24 hpi)	-2.5	0.01	-2.5	0.012433	ENSMMUG0000000 1044	AKT1	ENSMMUT000000 39437	7.2	0.000028	PI3K-Akt signaling pathway
					ENSMMUG0000000 8351	IKBKB	ENSMMUT000000 39043	2.1	0.000007	NF-kappa B signaling pathway
					ENSMMUG0000000 6613	MAP3K12	ENSMMUT0000000 9252	5.5	0.000283	MAPK signaling pathway
mml-miR-504-5p (24 hpi)	-2.1	0.02	-6.2	0.000007	ENSMMUG0000000 0803	STAT2	ENSMMUT0000000 1167	32.9	0.000005	Jak-STAT signaling pathway
					ENSMMUG000000 01819	ISG15	ENSMMUT000000 76502	4.8	0.006	RIG-I-like receptor signaling pathway
mml-miR-342-5p (12 hpi,24 hpi)	-3.4(12 hpi),-2.1(24 hpi)	0.007(12 hpi), 0.04(24 hpi)	-1.4(12 hpi), -16.2 (24 hpi)	0.2(12 hpi), 0.000002(24 hpi)	ENSMMUG0000000 1187	MAPK15	ENSMMUT0000000 1684	61.5 (12 hpi),14.7 (24 hpi)	0.009(12 hpi), 0.0002(24 hpi)	MAPK signaling pathway
mml-miR-365-1-5p (36 hpi)	-1.03	0.02	-3	0.004	ENSMMUG0000000 9349	ACACA	ENSMMUT000000 13072	-2.08	0.000263	AMPK signaling pathway
mml-miR-106b-3p (36 hpi)	1.1	0.004	2.7	0.0008	ENSMMUG000000 08634	BDNF	ENSMMUT000000 12071	-13	0.007	cAMP signaling pathway
mml-miR-299-3p (48 hpi)	1.3	0.0004	14	0.0003	ENSMMUG0000000 2952	LAMB2	ENSMMUT000000 74631	65.7	0.000097	PI3K-Akt signaling pathway
novel_366 (48 hpi)	4.7	0.003	8	0.02	ENSMMUG0000000 3364	BCL2A1	ENSMMUT0000000 4754	27.5	0.003	NF-kappa B signaling pathway

**TABLE 1b:** The other DE miRNAs without targets by bioinformatics or without significant targets by qPCR and not implicated in the signaling pathways, are marked by (−).

DE miRNA	Illumina RNA Sequencing		qPCR		Corresponding target gene ID	Corresponding target gene name	Corresponding target mRNA ID	qPCR		KEGG signaling pathway
	Log2 fold change	*P*-value	Log2 fold change	*P*-value				(log2 fold change)	*P*-value	
mml-let-7f-5p (0 hpi)	0.6	0.01	2.7	0.00004	ENSMMUG000000 22770	SERINC4	ENSMMUT000000 69670	45	0.001	-
mml-miR-99a-5p (0 hpi,6 hpi)	1.5(0 hpi), 1.7(6 hpi)	0.006(0 hpi), 0.02(6 hpi)	6.11 (0 hpi), 7.8 (6 hpi)	0.01(0 hpi), 0.001(6 hpi)	-	-	-	-		-
mml-miR-486-5p (6 hpi)	6.3	2.6259961 3001202E-13	18.8	0.001	-	-	-	-		-
mml-miR-132-3p (12 hpi)	0.95	0.04	4.8	0.03	ENSMMUG000000 22504	AZGP1	ENSMMUT000000 31648	19.3	0.006	-
novel_458 (12 hpi,36 hpi)	2.08(12 hpi), 2.1(36 hpi)	0.00008(12 hpi), 0.000003(36 hpi)	23.7 (12 hpi), 3.1(36 hpi)	0.0001(12 hpi), 0.001(36 hpi)	-	-	-	-		-
mml-miR-411-3p (12 hpi,48 hpi)	1.1(12 hpi), 0.9(48 hpi)	0.0009(12 hpi), 0.009(48 hpi)	2.3 (12 hpi), 2.4(48 hpi)	0.04(12 hpi), 0.002(48 hpi)	-	-	-	-		-
novel_356 (24 hpi,36 hpi)	5.6(24 hpi), 0.3(36 hpi)	0.01(24 hpi), 0.005(36 hpi)	3.5(24 hpi), 1.4(36 hpi)	N/A(24 hpi,36 hpi)	-	-	-	-		-
novel_66 (24 hpi,36 hpi)	3.08(24 hpi), 1.9(36 hpi)	0.001(24 hpi), 0.007(36 hpi)	13.4(24 hpi), 1(36 hpi)	0.0009(24 hpi), N/A(36 hpi)	-	-	-	-		-
mml-miR-451 (24 hpi)	2.7	0.03	2.5	0.04	ENSMMUG0000000 2776	ERAL1	ENSMMUT000000 03939	3.5	0.01	-
novel_352 (24 hpi)	3.03	0.03	2	0.05	-	-	-	-		-
mml-miR-197-3p (24 hpi)	-1.7	0.04	-2.5	0.005	ENSMMUG00000 004586	ORMDL3	ENSMMUT0000000 6483	5.7	0.003	-
					ENSMMUG000000 47699	TRBV16	ENSMMUT00000 054266	-2.3	0.05	-
					ENSMMUG0000000 4626	IFITM5	ENSMMUT0000000 6544	6.9	0.02	-
mml-miR-204-3p (24 hpi)	-2.5	0.01	-2.5	0.01	ENSMMUG0000000 5307	TRIM17	ENSMMUT000000 07474	11.7	0.0009	-
					ENSMMUG000000 05553	mTORC1 sensor	ENSMMUT000000 44103	15.5	0.002	-
					ENSMMUG000000 06441	OASL	ENSMMUT0000005 5549	8.9	0.00003	-
					ENSMMUG000000 07669	ISG20	ENSMMUT0000 0065082	9.8	0.003	-
					ENSMMUG0000000 9198	BCL6	ENSMMUT0000001 2862	-2.5	0.02	-
					ENSMMUG000000 05047	PHPT1	ENSMMUT000000 70130	31	0.00004	-
mml-miR-365-1-5p (36 hpi)	-1.03	0.02	-3	0.004	ENSMMUG00000 002389	HSPB6	ENSMMUT000000 03396	-3.2	0.0002	-
mml-miR-21-3p (36 hpi)	0.6	0.04	2.5	0.0004	ENSMMUG0000000 4486	COG6	ENSMMUT000000 06357	6.2	0.001	-
mml-miR-411-5p (36 hpi,48 hpi)	0.6(36 hpi), 1.1(48 hpi)	0.03(36 hpi), 0.00007(48 hpi)	3.5(36 hpi), 3.3(48 hpi)	0.003(36 hpi), 0.01(48 hpi)	-	-	-	-		-

**TABLE 2:** The expression levels (log2 fold changes) of common DE miRNAs, by Illumina sequencing and qPCR at the different time points of BRV infection.

miRNAs	Illumina RNA sequencing				qPCR			
	Log2 fold change		*P*-value		Log2 fold change		*P*-value	
	0 hpi	6 hpi	0 hpi	6 hpi	0 hpi	6 hpi	0 hpi	6 hpi
mml-miR-99a-5p	1.50333982	1.79094631	0.006	0.02	6.11	7.79	0.01	0.001
mml-miR-342-5p	12 hpi	24 hpi	12 hpi	24 hpi	12 hpi	24 hpi	12 hpi	24 hpi
	-3.4554665	-2.1650318	0.007	0.04	-1.4	-16.2	0.2	0.000002
mml-miR-411-3p	12 hpi	48 hpi	12 hpi	48 hpi	12 hpi	48 hpi	12 hpi	48 hpi
	1.14714287	0.93339313	0.0009	0.009	2.3	2.4	0.04	0.002
novel_356	24 hpi	36 hpi	24 hpi	36 hpi	24 hpi	36 hpi	24 hpi	36 hpi
	5.57138479	3.00636932	0.01	0.005	3.5	1.4	N/A	N/A
novel_66	3.08746627	1.96061132	0.001	0.007	13.5	1	0.0009	N/A
mml-miR-411-5p	36 hpi	48 hpi	36 hpi	48 hpi	36 hpi	48 hpi	36 hpi	48 hpi
	0.65336231	1.18032716	0.03	0.00007	3.5	3.3	0.003	0.01

## Discussion

The expression of the host miRNA and the differential expression induced by viral infection were used to investigate the virus–host interaction (Ghosh et al., 2009; Skalsky and Cullen, 2010). Some cellular miRNAs can mitigate viral replication either indirectly by regulating the host immune response or by directly targeting the virus (Slonchak et al., 2014). In contrast, other miRNAs can trigger viral replication by modulating the cellular environment (Jopling et al., 2005; Norman and Sarnow, 2010). Notwithstanding, there is no previous report on the relationship between BRV and cellular miRNAs. Therefore, in this study, the cellular DE miRNAs in response to BRV infection of MA-104 cells were identified using small miRNA sequencing of BRV-infected cells. We obtained 579 known miRNAs and 144 novel miRNAs and discovered 160 DE miRNAs in BRV-infected cells in comparison with the uninfected group, of which 95 were upregulated and 65 were downregulated. Bioinformatics analysis through miRanda and RNAhybrid revealed the target genes of the obtained DE miRNAs, and GO and KEGG analyses were performed to study the target mRNA functions and the biochemical metabolic pathways and signal transduction pathways (protein–protein interaction) of the target mRNAs involved in BP, CC, and MF. Moreover, real-time qPCR was used to validate the 23 DE miRNAs and 28 target mRNAs, which were harmonized with the deep sequencing and bioinformatics analyses. On the one hand, some of the validated miRNAs were upregulated [mml-let-7f-5p (0 hpi), mml-miR-99a-5p (0 and 6 hpi), mml-miR-486-3p (6 hpi), mml-miR-486-5p (6 hpi), mml-miR-127-3p (6 hpi), mml-miR-132-3p (12 hpi), mml-miR-369-5p (12 hpi), novel_458 (12 and 36 hpi), mml-miR-411-3p (12 and 48 hpi), mml-miR-451 (24 hpi), novel_352 (24 hpi), novel_356 (24 and 36 hpi), novel_66 (24 and 36 hpi), miR-21-3p (36 hpi), mml-miR-106b-3p (36 hpi), mml-miR-411-5p (36 and 48 hpi), mml-miR-299-3p (48 hpi), and novel_366 (48 hpi)]. On the other hand, a few others were downregulated [mml-miR-197-3p (24 hpi), mml-miR-204-3p (24 hpi), mml-miR-504-5p (24 hpi), mml-miR-365-1-5p (36 hpi), and mml-miR-342-5p (12 and 24 hpi)]. In other studies, miR-21-3p was found to be downregulated in the MA-104 cell line in response to both human RV strain ZTR-68 (P[8] G1) (Zhou et al., 2016) and strain Wa (Tian et al., 2017); surprisingly, this miRNA was upregulated in our study, which could be an indication of the different types of pathogenesis between the human and BRV strains. This deserves further investigation. It was demonstrated that EBV induces miR-21 during latency, linking this miRNA with viral persistence (Cameron et al., 2008). Some studies have reported the collaboration between cellular miRNAs and the biological processes and cellular signal transduction pathways. It was elucidated that miRNAs are closely linked to biological pathways such as stress response, proliferation, differentiation, apoptosis, and autophagy (Frankel and Lund, 2012). Previous research works have demonstrated that, despite autophagy being able to act as an antiviral mechanism, some viruses use this machinery in favor of viral replication (Shintani and Klionsky, 2004). Also, there were some speculations over the suppression of apoptosis being good for RV replication. For example, the inhibition of premature apoptosis by the NSP1-dependent PI3K3/Akt3/NF-κB pathway was suggested be favor viral growth of the simian RV strain SA11 (2–8 hpi) (Bagchi et al., 2010). Similarly, supportive proof has been reported for the PI3K/Akt/mTOR signaling pathway exerting an anti-autophagic role in SA11 rotavirus pathogenesis at 48 hpi, which in turn improves viral replication (Yin et al., 2018). Notwithstanding, another recent study has indicated that the decreased level of mTOR during early virus infection (4–6 hpi) could stimulate the autophagic pathway and help in the replication of RV (Mukhopadhyay et al., 2019). In addition, it was mentioned that the upregulation of let-7f-5p repressed several pro-apoptotic proteins (Tie et al., 2018). It was also confirmed that miR-99a-5p could target mTOR, while miR-486-3p and miR-486-5p were implicated in oncological and non-oncological conditions (ElKhouly et al., 2020). The downregulation of hsa-miR-127-3p could be the leading factor in the overexpression of type I interferon (IFN) (Wu et al., 2021). Besides, miR-132-3p and miR-369 suppressed the level of apoptosis, and miR-132-3p reduced the response of type I IFN to pave the way for influenza virus replication (Zhang F. et al., 2019; Qu et al., 2021; Wang J. et al., 2021). Moreover, miR-451-3p could mitigate apoptosis and autophagy (Tsai et al., 2018; Lv et al., 2021). Interestingly, in our study, mml-miR-127-3p, mml-miR-486-3p, and mml-miR-486-5p exhibited a surge in regulation at 6 hpi. mml-miR-99a-5p, mml-miR-132-3p, miR-369-5p, and mml-miR-451 likely play a role in cell survival and, thus, RV growth at 6, 12, and 24 hpi, which deserves further detailed study. Evidently, at 24 hpi, we observed that the DE miRNAs and their mRNAs were mainly of immune importance for defense against viral replication. On the contrary, the miR-197-3p and miR-504-5p in our study showed downregulation, with a previous report demonstrating that the inhibition of miR-197-3p represses cell apoptosis (Li et al., 2019). Additionally, the stimulation of miR-504 can enhance the apoptotic machinery (Hu L. et al., 2021). Together, it implies that the downregulation of miR-197-3p and miR-504-5p induced by BRV infection would suppress cell apoptosis to improve viral replication. Notably, the autophagic machinery was possibly operated *via* the collaboration between the downregulated miR-204 and the upregulated LC3-II protein (Xiao et al., 2011); therefore, the downregulation of miR-204-3p in our study could be implicated in the signaling transduction pathways for the virus strain replication. Because miR-21 has been reported as a pro-apoptotic miRNA that acts as a binding target of Bcl-2 (Jing et al., 2015), there was speculation around its participation as a pro-autophagic factor (Yang and Liang, 2015). As a result, the exact mechanism of the effect of both miR-204-3p and miR-21-3p on BRV replication needs further detailed investigation. At 36 hpi, our results showed the downregulation of mml-miR-365-1-5p and the upregulation of mml-miR-106b-3p. It has been mentioned that the upregulation of miR-365 promotes apoptosis *via* inactivation of the PI3K/AKT/mTOR pathway (Wang et al., 2020). Besides, miR-106b-3p has been used in the diagnosis of pancreatic cancer (Cao et al., 2016). Last but not least, mml-miR-299-3p and novel_366 exhibited a notable dramatic upregulation at 48 h, and it has been mentioned that miR-299-3p suppresses cell progression and induces apoptosis (Wang Z. et al., 2021). Evidently, mml-miR-342-5p was slightly downregulated at 12 hpi; however, it showed a marked downregulation at 24 hpi. Similarly, some novel DE miRNAs, such as novel_356 and novel_66, were upregulated at 24 hpi in comparison with 36 hpi, and novel_458 was also highly upregulated at 12 hpi compared to 36 hpi. Meanwhile, mml-miR99a-5p, mml-miR-411-3p, and mml-miR-411-5p exhibited the same upregulation levels at different time points (at 0 and 6 hpi, at 12 and 48 hpi, and at 36 and 48 hpi, respectively). Other reports have mentioned that miR-342-5p and miR-411-3p/5p were implicated in the formation of cancer (Lindholm et al., 2019; Zhang C. et al., 2019). In our study, real-time qPCR validated the bioinformatics results of the target mRNAs of the DE miRNAs that were implicated in the cellular biological processes and signaling pathways. Some of the upregulated targets are as follows: *SERINC4* (0 hpi); *PDPK1* (surge upregulation at 6 hpi); *AZGP1* (dramatic upregulation at 12 hpi); *ERAL1*, *ORMDL3*, *IFITM5*, *TRIM17*, and *mTORC1* (surge upregulation), *OASL*, *MAP3K12*, *ISG20*, and *PHPT1* (Janus superfamily, surge upregulation), *IKBKB*, *AKT1*, and *STAT2* (surge upregulation), and *ISG15* (all at 24 hpi); and *COG6* (at 36 hpi). *MAPK15* was dramatically upregulated at 12 hpi in comparison with 24 hpi. Additionally, *LAMB2* and *BCL2A1* both exhibited a surge in upregulation level at 48 hpi. The other targets were downregulated, as follows: *PARD6G* (at 6 hpi); *CD4* (at 12 hpi); *TRBV16*, *TSC2*, and *BCL6* (at 24 hpi); and *HSPB6*, *ACACA*, and *BDNF* (dramatic decrease at 36 hpi). Interestingly, *PDPK1* has been implicated in autophagosome biogenesis (Hu B. et al., 2021). It can modulate the autophagy pathway of our RV strain, which will need further detailed research, whereas *PARD6B* was shared in the cancer pathways (Cunliffe et al., 2012). *COG6* and *SERINC4* have been suggested to be inhibitors of HIV-1 replication (Liu et al., 2014; Qiu et al., 2020), while the expression of *AZGP1*, which has been reported to be associated with HPV status, was significantly high (Poropatich et al., 2019). *ERAL1* suppressed RNA virus infection by facilitating RIG-I-like receptor signaling (Li et al., 2021), while *ORMDL3* was reported to be regulated by IRF-3 as a result of RSV infection by direct binding to the promoter of *ORMDL3* (Wang et al., 2017). Evidently, *STAT2*, as a transcription regulatory factor, could increase the levels of the interferon-stimulated gene (ISG) and IFN transcripts in response to RV, establishing an antiviral state (Angel et al., 2012). The IFITM family (interferon-induced genes) was found to respond to type I and II IFNs (Yánez et al., 2020), and OASL, a family member of ISGs, was documented as one of the key contributors in the control of antiviral innate immunity, specially *ISG20* and *ISG15*, which were reported to be induced or regulated by both type I and II IFNs (Zhu et al., 2015; Zheng et al., 2017). Moreover, *TRIM17* can not only promote the degradation of the anti-apoptotic protein MCL1 but also prevent the ubiquitination of other proteins and stabilize them by binding to other TRIM proteins and inhibiting their E3 ubiquitin ligase activity. This dual action confers several pivotal roles to *TRIM17* in crucial cellular processes such as apoptosis and autophagy (Basu-Shrivastava et al., 2021). On the contrary, the MAPK family members have a close relationship with other viral signaling pathways, such as NF-κB, PI3K/Akt, and Janus kinase/signal transducer and activator of transcription (JAK-STAT) (Chander et al., 2021). Notwithstanding, the transcription factor and proto-oncogene B-cell CLL/lymphoma 6 (*BCL6*) could mediate the transcriptional repression of LITAF, which may inhibit autophagy in B cells during the germinal center reaction (Bertolo et al., 2013). *HSPB6* was reported to suppress the MAPK family, including JNK and the PI3K/Akt pathway (Matsushima-Nishiwaki et al., 2011, 2013), and the downregulated *ACACA* curbed prostate cancer growth (Zhang et al., 2021). Last but not least, the BCL2 protein was reported as an anti-apoptotic factor via its regulation of NF-κB target gene that exerts important pro-survival functions (Vogler, 2012).

## Conclusion

**FIGURE 10:**
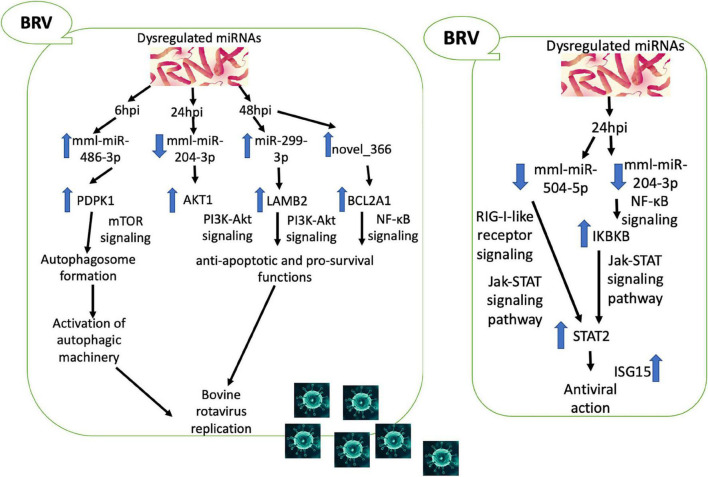
Predicted crosstalk between the differentially expressed (DE) microRNAs (miRNAs), target messenger RNAs (mRNAs), and the signaling pathways during bovine rotavirus (BRV) infection.

In conclusion, our study elucidated a novel approach to reveal the miRNAs of the MA-104 cell line induced by BRV infection and the crosstalk between the DE miRNAs, target mRNAs, and the signaling pathways during BRV infection. At 6 hpi, BRV upregulated mml-miR-486-3p, which may, in turn, elevate the expression of target *PDPK1*, implicated in autophagosome formation (mTOR signaling pathway) in favor of viral replication. At 24 hpi, BRV downregulated mml-miR-204-3p, which may, in turn, upregulate its target *AKT1*. At 48 hpi, BRV upregulated both mml-miR-299-3p and novel_366, resulting in the upregulation of the targets *LAMB2* and *BCL2A1*, respectively. These results (at 24 and 48 hpi) exerted anti-apoptotic and pro-survival functions *via* the PI3K/Akt and NF-κB signaling pathways, improving viral replication. On the other hand, at 24 hpi, the host immune system was activated against viral replication through the downregulation of both mml-miR-204-3p, which activated *IKBKB* (NF-κB signaling), and mml-miR-504-5p (RIG-I-like receptor signaling pathway), which triggered *STAT2* (JAK-STAT signaling pathway), providing antiviral status (Figure 10). Thus, it could be of value to reduce the gap in BRV pathogenesis and to determine potential targets for the development of novel antiviral drugs and vaccines.

## Data Availability Statement

The analyzed data that supports the findings of this study are available as the Supplementary Material of this article. The data that support the findings of this study were deposited in GEO data repository under accession GSE196536, using the following contact links: (https://www.ncbi.nlm.nih.gov/geo/query/acc.cgi?acc=GSE196536) and (https://www.ncbi.nlm.nih.gov/geo/info/linking.html). And our data are available from the corresponding author upon reasonable request.

## Author Contributions

GE designed and performed the practical experiments and drafted the manuscript. YC, CH, JC, and XC coordinated the project. AG contributed to the supervision and revision of the manuscript. All authors contributed to the article and approved the submitted version.

## Conflict of Interest

The authors declare that the research was conducted in the absence of any commercial or financial relationships that could be construed as a potential conflict of interest.

## Publisher’s Note

All claims expressed in this article are solely those of the authors and do not necessarily represent those of their affiliated organizations, or those of the publisher, the editors and the reviewers. Any product that may be evaluated in this article, or claim that may be made by its manufacturer, is not guaranteed or endorsed by the publisher.

## Footnotes

1
http://www.mirbase.org/

2
https://genie.weizmann.ac.il/pubs/mir07/mir07_prediction.html

3
http://bibiserv.techfak.uni-bielefeld.de/rnahybrid/

4
http://www.geneontology.org/

5
http://kobas.cbi.pku.edu.cn/help.do
