# Supplementary material for: MicroRNA Profile of MA-104 Cell Line Associated With the Pathogenesis of Bovine Rotavirus Strain Circulated in Chinese Calves
Source: Front Microbiol. 2022 Apr 11;13:854348. doi: 10.3389/fmicb.2022.854348 (PMC9062783; doi:10.3389/fmicb.2022.854348)
Supplement: Supplementary file 1 [file Data_Sheet_1.ZIP › Tables/Table 1.docx]

| DE miRNA | Illumina RNA Sequencing | | qPCR | | Corresponding target gene ID | Corresponding target gene name | Corresponding target mRNA ID | qPCR | | KEGG signaling pathway |
| --- | --- | --- | --- | --- | --- | --- | --- | --- | --- | --- |
|  | (log2fold change) | P value | (log2fold change) | P value |  |  |  | (log2 fold change) | P value |  |
| mml-miR-486-3p (6hpi) | 6.25 | 3.80355330136931E-13 | 11.5 | 0.000011 | ENSMMUG00000011459 | PDPK1 | ENSMMUT00000058708 | 42 | 0.02 | mTOR signaling pathway |
| mml-miR-127-3p (6hpi) | 2.4 | 0.00357868185018267 | 242.5 | 0.000002 | ENSMMUG00000039029 | PARD6G | ENSMMUT00000067376 | -2.3 | 0.02 | Rap1 signaling pathway |
| mml-miR-369-5p (12hpi) | 1.2 | 0.005 | 4.2 | 0.003 | ENSMMUG00000013202 | CD4 | ENSMMUT00000018518 | -15.3 | 0.000719 | Primary immune difficiency, T cell receptor signaling pathway |
| mml-miR-197-3p (24hpi) | -1.7 | 0.04 | -2.5 | 0.005337 | ENSMMUG00000002808 | TSC2 | ENSMMUT00000059008 | -2.05 | 0.04 | mTOR signaling pathway |
| mml-miR-204-3p (24hpi) | -2.5 | 0.01 | -2.5 | 0.012433 | ENSMMUG00000001044 | AKT1 | ENSMMUT00000039437 | 7.2 | 0.000028 | PI3K-Akt signaling pathway |
|  |  |  |  |  | ENSMMUG00000008351 | IKBKB | ENSMMUT00000039043 | 2.1 | 0.000007 | NF-kappa B signaling pathway |
|  |  |  |  |  | ENSMMUG00000006613 | MAP3K12 | ENSMMUT00000009252 | 5.5 | 0.000283 | MAPK signaling pathway |
| mml-miR-504-5p (24hpi) | -2.1 | 0.02 | -6.2 | 0.000007 | ENSMMUG00000000803 | STAT2 | ENSMMUT00000001167 | 32.9 | 0.000005 | Jak-STAT signaling pathway |
|  |  |  |  |  | ENSMMUG00000001819 | ISG15 | ENSMMUT00000076502 | 4.8 | 0.006 | RIG-I-like receptor signaling pathway |
| mml-miR-342-5p (12hpi,24hpi) | -3.4(12hpi),-2.1(24hpi) | 0.007(12hpi),0.04(24hpi) | -1.4(12hpi), -16.2 (24hpi) | 0.2(12hpi), 0.000002(24hpi) | ENSMMUG00000001187 | MAPK15 | ENSMMUT00000001684 | 61.5 (12hpi), 14.7 (24hpi) | 0.009(12hpi), 0.0002(24hpi) | MAPK signaling pathway |
| mml-miR-365-1-5p (36hpi) | -1.03 | 0.02 | -3 | 0.004 | ENSMMUG00000009349 | ACACA | ENSMMUT00000013072 | -2.08 | 0.000263 | AMPK signaling pathway |
| mml-miR-106b-3p (36hpi) | 1.1 | 0.004 | 2.7 | 0.0008 | ENSMMUG00000008634 | BDNF | ENSMMUT00000012071 | -13 | 0.007 | cAMP signaling pathway |
| mml-miR-299-3p (48hpi) | 1.3 | 0.0004 | 14 | 0.0003 | ENSMMUG00000002952 | LAMB2 | ENSMMUT00000074631 | 65.7 | 0.000097 | PI3K-Akt signaling pathway |
| novel_366 (48hpi) | 4.7 | 0.003 | 8 | 0.02 | ENSMMUG00000003364 | BCL2A1 | ENSMMUT00000004754 | 27.5 | 0.003 | NF-kappa B signaling pathway |

**Table 1** a. The selected DE miRNAs and their corresponding genes, corresponding target mRNA transcripts and the siganling pathways they implicated in.

| DE miRNA | Illumina RNA Sequencing | | qPCR | | Corresponding target gene ID | Corresponding target gene name | Corresponding target mRNA ID | qPCR | | KEGG signaling pathway |
| --- | --- | --- | --- | --- | --- | --- | --- | --- | --- | --- |
|  | Log2 fold change | P value | Log2 fold change | P value |  |  |  |  |  |  |
|  |  |  |  |  |  |  |  | (log2 fold change) | P value |  |
| mml-let-7f-5p (0hpi) | 0.6 | 0.01 | 2.7 | 0.00004 | ENSMMUG00000022770 | SERINC4 | ENSMMUT00000069670 | 45 | 0.001 | - |
| mml-miR-99a-5p (0hpi,6hpi) | 1.5(0hpi),1.7(6hpi) | 0.006(0hpi)0.02(6hpi) | 6.11 (0hpi), 7.8 (6hpi) | 0.01(0hpi),0.001(6hpi) | - | - | - | - |  | - |
| mml-miR-486-5p (6hpi) | 6.3 | 2.62599613001202E-13 | 18.8 | 0.001 | - | - | - | - |  | - |
| mml-miR-132-3p (12hpi) | 0.95 | 0.04 | 4.8 | 0.03 | ENSMMUG00000022504 | AZGP1 | ENSMMUT00000031648 | 19.3 | 0.006 | - |
| novel_458 (12hpi,36hpi) | 2.08(12hpi),2.1(36hpi) | 0.00008(12hpi), 0.000003(36hpi) | 23.7 (12hpi), 3.1(36hpi) | 0.0001(12hpi), 0.001(36hpi) | - | - | - | - |  | - |
| mml-miR-411-3p (12hpi,48hpi) | 1.1(12hpi),0.9(48hpi) | 0.0009(12hpi).0.009(48hpi) | 2.3 (12hpi), 2.4(48hpi) | 0.04(12hpi), 0.002(48hpi) | - | - | - | - |  | - |
| novel_356 (24hpi,36hpi) | 5.6(24hpi), 0.3(36hpi) | 0.01(24hpi),0.005(36hpi) | 3.5(24hpi), 1.4(36hpi) | N/A(24hpi,36hpi) | - | - | - | - |  | - |
| novel_66 (24hpi,36hpi) | 3.08(24hpi),1.9(36hpi) | 0.001(24hpi).0.007(36hpi) | 13.4(24hpi),1(36hpi) | 0.0009(24hpi),N/A(36hpi) | - | - | - | - |  | - |
| mml-miR-451 (24hpi) | 2.7 | 0.03 | 2.5 | 0.04 | ENSMMUG00000002776 | ERAL1 | ENSMMUT00000003939 | 3.5 | 0.01 | - |
| novel_352 (24hpi) | 3.03 | 0.03 | 2 | 0.05 | - | - | - | - |  | - |
| mml-miR-197-3p (24hpi) | -1.7 | 0.04 | -2.5 | 0.005 | ENSMMUG00000004586 | ORMDL3 | ENSMMUT00000006483 | 5.7 | 0.003 | - |
|  |  |  |  |  | ENSMMUG00000047699 | TRBV16 | ENSMMUT00000054266 | -2.3 | 0.05 | - |
|  |  |  |  |  | ENSMMUG00000004626 | IFITM5 | ENSMMUT00000006544 | 6.9 | 0.02 | - |
| mml-miR-204-3p (24hpi) | -2.5 | 0.01 | -2.5 | 0.01 | ENSMMUG00000005307 | TRIM17 | ENSMMUT00000007474 | 11.7 | 0.0009 | - |
|  |  |  |  |  | ENSMMUG00000005553 | mTORC1 sensor | ENSMMUT00000044103 | 15.5 | 0.002 | - |
|  |  |  |  |  | ENSMMUG00000006441 | OASL | ENSMMUT00000055549 | 8.9 | 0.00003 | - |
|  |  |  |  |  | ENSMMUG00000007669 | ISG20 | ENSMMUT00000065082 | 9.8 | 0.003 | - |
|  |  |  |  |  | ENSMMUG00000009198 | BCL6 | ENSMMUT00000012862 | -2.5 | 0.02 | - |
|  |  |  |  |  | ENSMMUG00000005047 | PHPT1 | ENSMMUT00000070130 | 31 | 0.00004 | - |
| mml-miR-365-1-5p (36hpi) | -1.03 | 0.02 | -3 | 0.004 | ENSMMUG00000002389 | HSPB6 | ENSMMUT00000003396 | -3.2 | 0.0002 | - |
| mml-miR-21-3p (36hpi) | 0.6 | 0.04 | 2.5 | 0.0004 | ENSMMUG00000004486 | COG6 | ENSMMUT00000006357 | 6.2 | 0.001 | - |
| mml-miR-411-5p (36hpi,48hpi) | 0.6(36hpi),1.1(48hpi) | 0.03(36hpi), 0.00007(48hpi | 3.5(36hpi), 3.3(48hpi) | 0.003(36hpi), 0.01(48hpi) | - | - | - | - |  | - |

Table 1b. The other DE miRNAs without targets by bioinformatics or without significant targets by qPCR and not implicated in the signaling pathways, are marked by (-).
