# Supplementary material for: MicroRNA Profile of MA-104 Cell Line Associated With the Pathogenesis of Bovine Rotavirus Strain Circulated in Chinese Calves
Source: Front Microbiol. 2022 Apr 11;13:854348. doi: 10.3389/fmicb.2022.854348 (PMC9062783; doi:10.3389/fmicb.2022.854348)
Supplement: Supplementary file 1 [file Data_Sheet_1.ZIP › Tables/Table 2.docx]

| miRNAs | Illumina RNA sequencing | | | | qPCR | | | |
| --- | --- | --- | --- | --- | --- | --- | --- | --- |
|  | Log2 fold change | | P value | | Log2 fold change | | P value | |
|  | 0hpi | 6hpi | 0hpi | 6hpi | 0hpi | 6hpi | 0hpi | 6hpi |
| mml-miR-99a-5p | 1.50333982 | 1.79094631 | 0.006 | 0.02 | 6.11 | 7.79 | 0.01 | 0.001 |
| mml-miR-342-5p | 12hpi | 24hpi | 12hpi | 24hpi | 12hpi | 24hpi | 12hpi | 24hpi |
|  | -3.4554665 | -2.1650318 | 0.007 | 0.04 | -1.4 | -16.2 | 0.2 | 0.000002 |
| mml-miR-411-3p | 12hpi | 48hpi | 12hpi | 48hpi | 12hpi | 48hpi | 12hpi | 48hpi |
|  | 1.14714287 | 0.93339313 | 0.0009 | 0.009 | 2.3 | 2.4 | 0.04 | 0.002 |
| novel_356 | 24hpi | 36hpi | 24hpi | 36hpi | 24hpi | 36hpi | 24hpi | 36hpi |
|  | 5.57138479 | 3.00636932 | 0.01 | 0.005 | 3.5 | 1.4 | N/A | N/A |
| novel_66 | 3.08746627 | 1.96061132 | 0.001 | 0.007 | 13.5 | 1 | 0.0009 | N/A |
| mml-miR-411-5p | 36hpi | 48hpi | 36hpi | 48hpi | 36hpi | 48hpi | 36hpi | 48hpi |
|  | 0.65336231 | 1.18032716 | 0.03 | 0.00007 | 3.5 | 3.3 | 0.003 | 0.01 |

**Table 2.** The expression levels (log2 fold changes) of common DE miRNAs, by Illumina sequencing and qPCR at the different time points of BRV infection.
