# Supplementary material for: MicroRNA Profile of MA-104 Cell Line Associated With the Pathogenesis of Bovine Rotavirus Strain Circulated in Chinese Calves
Source: Front Microbiol. 2022 Apr 11;13:854348. doi: 10.3389/fmicb.2022.854348 (PMC9062783; doi:10.3389/fmicb.2022.854348)
Supplement: Supplementary file 3 [file Data_Sheet_3.PDF]

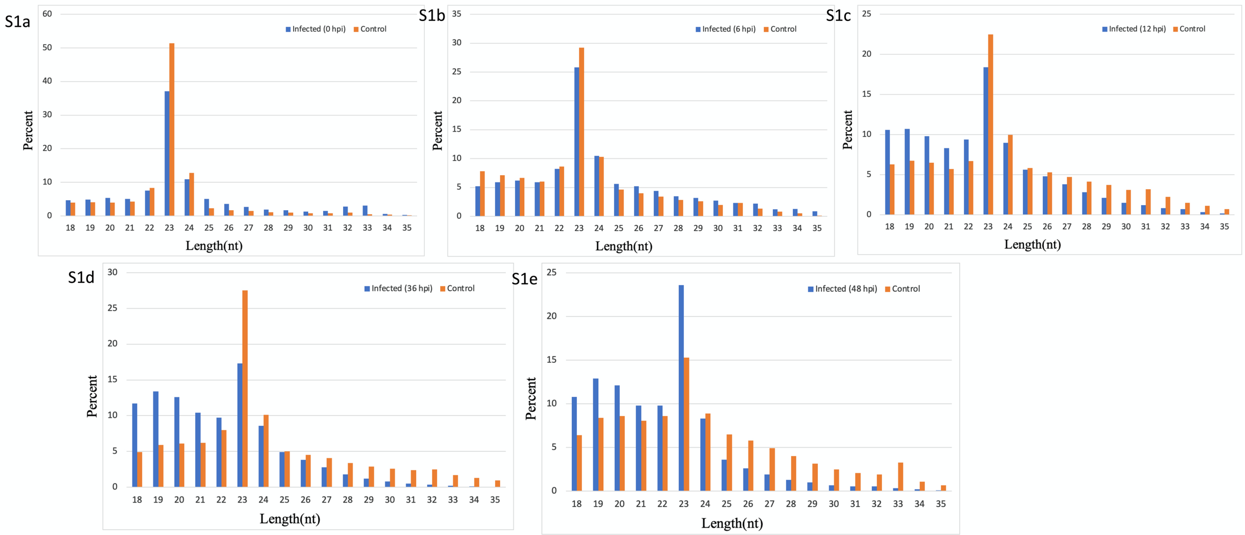

Supplementary Fig. S1a-S1e. The length distribution statistics of total sRNA segments obtained at (0 hpi, 6 hpi, 12 hpi, 36 hpi, 48 hpi). The length of reads in proportional to the reads of that length, 23nt is the main observed length.

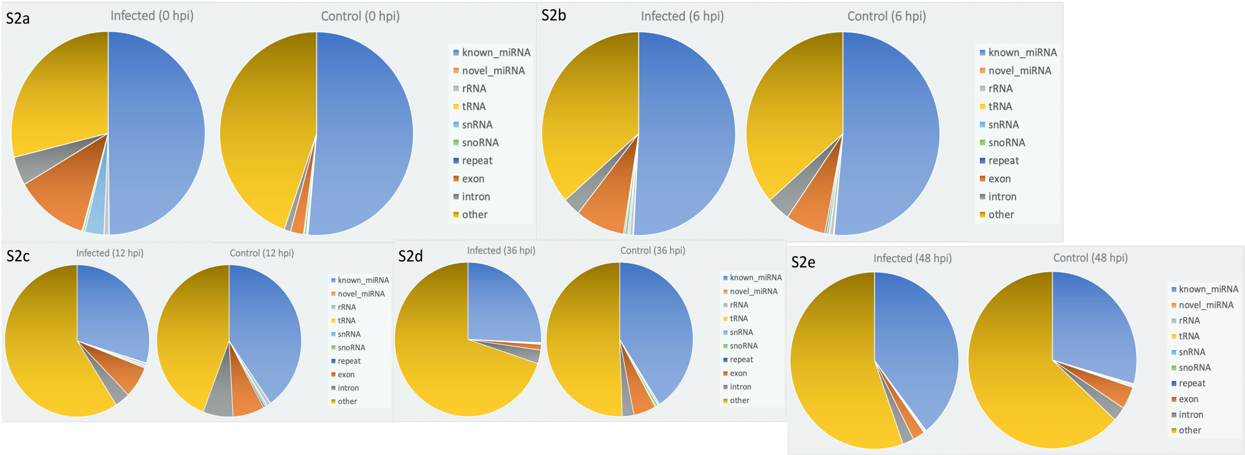

Supplementary Fig. S2a-S2e. The pie charts showed total reads of distribution statistics of small RNA and their categories at (0 hpi, 6 hpi, 12 hpi, 36 hpi, 48 hpi). Known miRNAs, novel miRNAs, rRNA, tRNA, snRNA, snoRNA, repeats, exons, introns and other.

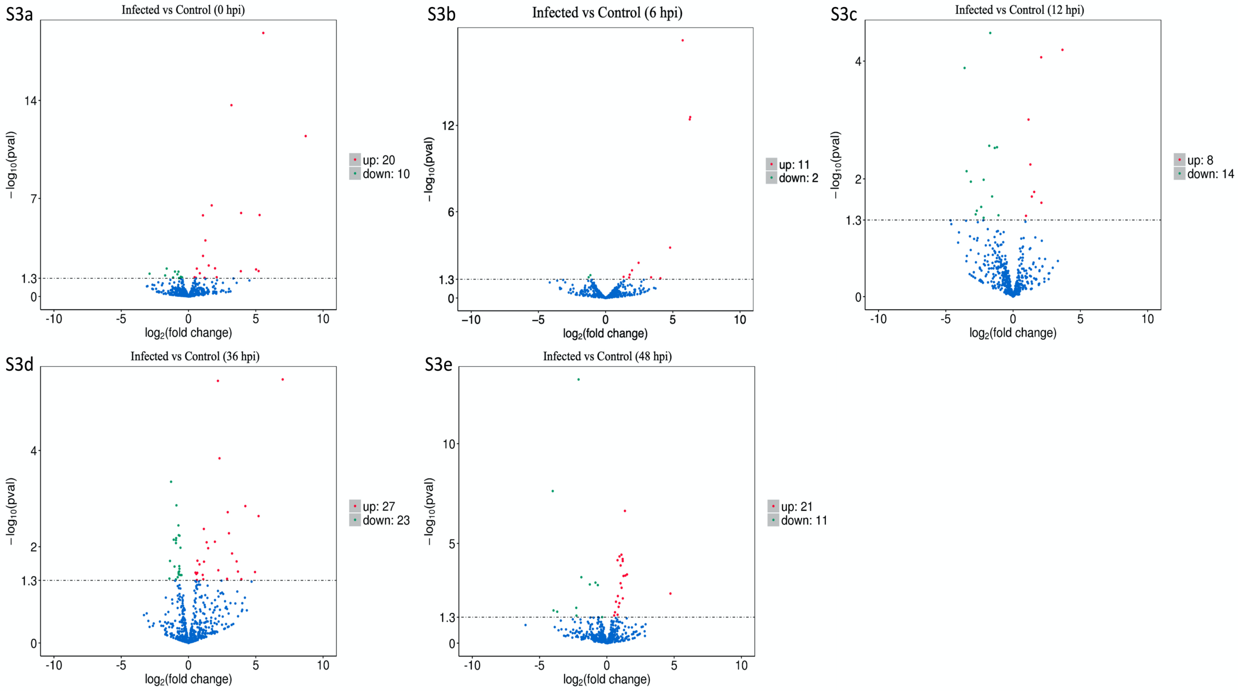

Supplementary Fig. S3a-S3e. Volcano chart represents the expression change of miRNA between virus treated and normal non treated cells (0 hpi, 6 hpi, 12 hpi, 36 hpi, 48 hpi). The x-axis shows the log2 fold change and y-axis shows the −log10 (q-value). The horizontal dotted lines in the figure correspond to qvalue (default) or adjusted P-value (FDR) =0.05 significant difference threshold. The significant difference threshold of log2 fold change >1, and significant difference threshold of adjusted P-value (FDR) =0.05. The up-regulated miRNAs are represented by red dots, down-regulated miRNAs are represented by green dots, and blue dots are miRNAs with no significant changes.
